# Structural and functional implications of positive selection at the primate angiogenin gene

**DOI:** 10.1186/1471-2148-7-167

**Published:** 2007-09-20

**Authors:** Daniel S Osorio, Agostinho Antunes, Maria J Ramos

**Affiliations:** 1 REQUIMTE, Departamento de Química, Faculdade de Ciências, Universidade do Porto, Rua do Campo Alegre, 687, 4169-007 Porto, Portugal; 2 INSERM UMR S 787-Groupe Myologie, Faculté de Médecine – Pitié-Salpétrière, UPMC Paris VI, 105 bd. de l'Hôpital, 75634, Paris Cedex 13, France; 3 CIMAR, Centro Interdisciplinar de Investigação Marinha e Ambiental, Universidade do Porto, Rua dos Bragas, 177, 4050-123 Porto, Portugal

## Abstract

**Background:**

Angiogenesis, the formation of new blood vessels, is a primordial process in development and its dysregulation has a central role in the pathogenesis of many diseases. Angiogenin (ANG), a peculiar member of the RNase A superfamily, is a potent inducer of angiogenesis involved in many different types of cancer, amyotrophic lateral sclerosis and also with a possible role in the innate immune defense. The evolutionary path of this family has been a highly dynamic one, where positive selection has played a strong role. In this work we used a combined gene and protein level approach to determine the main sites under diversifying selection on the primate ANG gene and analyze its structural and functional implications.

**Results:**

We obtained evidence for positive selection in the primate ANG gene. Site specific analysis pointed out 15 sites under positive selection, most of which also exhibited drastic changes in amino acid properties. The mapping of these sites in the ANG 3D-structure described five clusters, four of which were located in functional regions: two in the active site region, one in the nucleolar location signal and one in the cell-binding site. Eight of the 15 sites under selection in the primate ANG gene were highly or moderately conserved in the RNase A family, suggesting a directed event and not a simple consequence of local structural or functional permissiveness. Moreover, 11 sites were exposed to the surface of the protein indicating that they may influence the interactions performed by ANG.

**Conclusion:**

Using a maximum likelihood gene level analysis we identified 15 sites under positive selection in the primate ANG genes, that were further corroborated through a protein level analysis of radical changes in amino acid properties. These sites mapped onto the main functional regions of the ANG protein. The fact that evidence for positive selection is present in all ANG regions required for angiogenesis may be a good indication that angiogenesis is the process under selection. However, other possibilities to be considered arise from the possible involvement of ANG in innate immunity and the potential influence or co-evolution with its interacting proteins and ligands.

## Background

Angiogenesis, the physiological process involving the growth of new blood vessels, is a primordial process in development. The complex network of interactions between pro- and anti-angiogenic regulators dictates that any imbalance in this process can lead to disease. Indeed, angiogenesis plays a central role in the pathophysiology of cancer, rheumatoid arthritis, diabetic retinopathy and several heart diseases (reviewed in [1]). Angiogenin (ANG), a potent in vivo inducer of angiogenesis, was first isolated in a systematic search for angiogenic factors secreted by human HT-29 colon adenocarcinoma cells [2]. Its increased expression was subsequently documented in different tumors and in several instances correlated with the disease progression and aggressiveness (reviewed in [3]). The use of antibodies [4-7], antisense targeting [8] and inhibitors [9,10] has proven useful in inhibiting the establishment, progression and metastasis of tumors in mouse models, thereby establishing ANG as a promising target for anticancer therapy. Furthermore, ANG was shown to have *in vitro *antimicrobial [11] and antiviral [12] effects, raising the possibility of its implication in the innate immune system. Recently, ANG mutations were described in amyotrophic lateral patients [13], constituting the second angiogenic factor implicated in this disease [14].

The human ANG gene comprises a single exon flanked by small UTRs and codes for a 14 kDa polypeptide. The protein is synthesized with a 24 amino acid signal peptide that is cleaved to produce a mature form with 123 amino acids. Sequence analysis revealed its homology to the Ribonuclease A (RNase A) superfamily, where it was included and classified as RNase 5 [15,16]. ANG has three main distinctive features when compared to the family archetype bovine RNase A: (1) the characteristic ribonuclease activity towards poly-, di- and cyclic nucleotides is 10^4^-10^6 ^lower and its enzymatic specificity is also different [17]; (2) the region between residues 58–70 appears to constitute a 'cell-binding site', independent from the active site [18,19], probably involved primarily in protein-protein interactions; and (3) the region 31–35 constitutes a nucleolar localization signal [20]. All of these features are essential to the angiogenic activity as shown by directed mutagenesis experiments [20-23].

The RNase superfamily has a highly dynamic evolutionary history, in which ANG occupies an important position. The fact that only ANG/RNase 5-like ribonucleases are found in non-mammalian vertebrates has led to the hypothesis that the RNase ancestral was structurally similar to ANG. This ancestral enzyme was most likely involved in host-pathogen interactions and did not possess an angiogenic activity [24]. A gene expansion occurred before the divergence between placental and marsupial mammals, followed by a process of differential gene duplication and retention between different orders of the placental mammals, which resulted in the present inter-species diversity of the RNase superfamily [25].

Diversifying (positive) selection had a strong influence in the RNase A superfamily evolutionary pathway: eosinophil RNases, EDN and ECP, are among the most rapidly evolving coding sequences in primates [26], as are the paralogous rodent eosinophil associated ribonucleases (EARs) [27]. ANG genes suffered a rapid expansion in rodents [28] – 6 genes and 3 pseudogenes in the mouse genome, 2 genes in the rat genome – as the result of positive selection and gene sorting. ANG was also previously shown to be under the effect of diversifying selection in primates [29,30]. All primate species analyzed thus far possess a single gene for ANG except for *Pygathrix nemaeus *(Asian Douc Langur), in which the ANG gene appears to have pseudogenized [31].

In this work we assessed the impacts of positive selection on the primate ANG gene using: (1) a gene level evaluation of the non-synonymous/synonymous ratio (dN/dS) and (2) a protein level evaluation of radical changes in amino acid properties. Several sites under positive selection were detected in the different functional regions of ANG and the effects in its structure and function have been analyzed.

## Results and discussion

### Phylogenetic analyses

**Figure 1:**
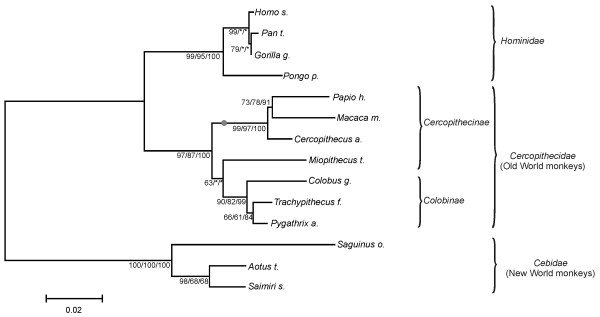
**Phylogenetic tree for the primate ANG sequences used**. Neighbor-Joining phylogenetic tree of the primate species analyzed. The bootstrap values for different methodologies are indicated bellow each branch (NJ/ML/BY). The symbol (*) indicates distinct topological arrangements. The symbol (•) indicates the branch with a significant LRT in the PAML branch analysis.

Neighbor-Joining (NJ), maximum-likelihood (ML) and Bayesian (BAY) tree reconstructions of the primate ANG coding sequences (table 1) presented similar overall topologies (figure 1). ML and BAY trees were topologically identical, the main difference relatively to the NJ tree being the unresolved Homo/Pan/Gorilla trichotomy and the positioning of *Miopithecus talapoin*. Overall, tree topologies were mostly coherent with the accepted phylogeny of primates. The small differences detected are not surprising since the gene tree does not necessarily reflect the species tree [32].

**Table 1:** Species and sequence reference number used in this study

**Common Name**	**Species**	**GenBank Reference**
Human	*Homo sapiens*	NM_001145
Chimpanzee	*Pan troglodytes*	NM_001009159
Gorilla	*Gorilla gorilla*	AF441662
Orangutan	*Pongo pygmaeus*	AF441663
Baboon	*Papio hamadryas*	AF441666
Rhesus macaque	*Macaca mulatta*	AF441667
Vervet monkey	*Cercopithecus aethiops*	AF441664
Talapoin monkey	*Miopithecus talapoin*	AF441665
Tonkin snub-nosed monkey	*Pygathrix avunculus*	AY221132
François's leaf monkey	*Trachypithecus francoisi*	AY221129
Mantled guereza	*Colobus guereza*	AY221128
South American squirrel monkey	*Saimiri sciureus*	AF441670
Cotton-top tamarin	*Saguinus oedipus*	AF441668
Northern night monkey	*Aotus trivirgatus*	AF441669

### Positive selection analyses

We first analyzed primate ANG genes for signatures of positive selection using PAML [33]. This software uses a maximum-likelihood approach to determine the non-synonymous to synonymous rate ratio (ω), also designated Ka/Ks or dN/dS, a widely used measure to detect departures from neutrality as indicators of selective pressures on protein coding genes. A ω > 1 indicates positive selection, whereas ω = 1 indicates neutrality and ω < 1 is indicative of negative selection. Branch models, where ω is permitted to vary between lineages, were first tested. The simplest model (one ratio), a very strict model allowing a single ω for all branches, obtained a ω = 1.2934, providing a good support for positive selection in the primate ANG. Two ω ratios models and a free ω ratios model where also tested (see additional files 1 and 2) and LRTs performed against adequate null models. Several branches presented a ω ratio above one, but only the two-ratio model for the branch including *Papio hamadryas*, *Macaca mulatta *and *Cercopithecus aethiops *(figure 1) presented a significant LRT. We then tested three nested pairs of site models that allow ω variation between codons: one admitting no positive selection (restricting ω ≤ 1) and another admitting positive selection (M1a vs. M2a, M7 vs. M8 and M8a vs. M8). Both positive selection admitting models, M2a and M8, presented a significantly better fit to the data than their neutral counterparts M1a, M7 and M8a (table 2). The slightly more conservative model M2 detected 41% of sites under positive selection (ω = 3.10), whereas M8 detected 49% of sites under positive selection (ω = 2.88), indicating with a high degree of confidence that the ANG gene is under positive selection. These results were consistent across all the tree reconstruction methods used. Posterior Bayesian analysis through a Bayes Empirical Bayes (BEB) methodology [34] allowed the determination of several amino acid sites under positive selection. M2 detected four sites with ω > 1 having a posterior probability (PP) above 0.95 and four above 0.99, while M8 detected nine sites above 0.95 and seven above 0.99. Either of the models detected several other sites with a PP above 0.9. Similar results were obtained across all tree topologies, with the exception of site 52 that only had a strong support in the NJ topology. Since M2a is more conservative and M8 more prone to false positives [35], we adopted an empiric threshold to consider candidate sites under positive selection: a PP above 0.95 for M8 and simultaneously above 0.90 for M2. Fifteen sites were above the defined threshold (table 3).

**Table 2:** Likelihood ratio tests for PAML site models

**Model**	**Parameters**	**lnL**	**2ΔlnL (LRT)**	
M0	ω = 1.2934	-1632.9385	Na	

M1a	*p*_0 _= 0.38665	-1614.8048	M1a vs. M2a	
	p_1 _= 0.61335		27.2618	
	ω_0 _= 0.00000			
	ω_1 _= 1.00000		(*p *= 1.2027E-06)	
	
M2a	*p*_0 _= 0.40009	-1601.1739		
	p_1 _= 0.18886			
	p_2 _= 0.41105			
	ω_0 _= 0.00000			
	ω_1 _= 1.00000			
	ω_2 _= 3.09729			

M7	*p *= 0.00750	-1614.8327	M7vs M8	M8a vs. M8
	*q *= 0.00500			
	
M8	*p*_0 _= 0.51176	-1601.3690	26.9274	26.8716
	p_1 _= 0.48824		(*p *= 1.4216E-06)	(*p *= 2.1743E-07)
	*p *= 0.19739			
	*q *= 1.36510			
	ω = 2.88352			
	
M8a	*p*_0 _= 0.38665	-1614.8048		
	p_1 _= 0.61335			
	p = 0.00500			
	q = 69.77288			
	ω = 1.00000			

**Table 3:** Sites under positive selection on the primate ANG gene

**Sites**		**PAML ω/PP (BEB)**			**TreeSAAP properties**					
Codo n	Amino acid	M2a	M8	**Total**	Chemical		Structural		Other	

**28**	**4 Ser**	**3.376 ± 0.53 0.999**	**3.210 ± 0.518 1.000**	**9**	**4**	***R***_***F***_***, H***_***nc***_**, *E***_***sm***_**,*R***_***a***_	**5**	***F*, *P***_***c***_**,*B***_***I***_**, *α ***_***n***_**, *P***	**0**	**----**
31	7 Thr	3.246 ± 0.735 0.946	3.158 ± 0.610 0.980	4	3	*R*_*F*_, *pK'*, *H*	1	*α*_*n*_	0	----
32	8 His	3.245 ± 0.73 0.943	3.166 ± 0.590 0.982	1	0	---------	1	*α*_*c*_	0	----
35	11 Thr	3.369 ± 0.547 0.996	3.208 ± 0.521 0.999	3	0	---------	3	*P*_β_, *K*^0^, *α*_*c*_	0	----
56	32 Arg	3.246 ± 0.73 0.943	3.157 ± 0.610 0.979	5	2	*pH*_*i*_, *p*	3	*F*, *α*_*c*_, *P*	0	----
**58**	**34 Gly**	**3.229 ± 0.740 0.936**	**3.160 ± 0.607 0.979**	**9**	**7**	***B***_***r***_**, *R***_***F***_**, *E***_***l***_**, *H***_***nc***_***, R***_***a***_**, *H***_***p***_**, *E***_***t***_	**2**	***N***_***s***_**, *P***_**β**_	**0**	**----**
**65**	**41 Asp**	**3.263 ± 0.709 0.950**	**3.173±0.587 0.984**	**7**	**3**	***pK'*, *P***_***r***, _***p***	**3**	***K***^**0**^**, *F*, *α ***_***c***_	**1**	***M***_***w***_
75	51 Arg	3.349 ± 0.584 0.986	3.202 ± 0.534 0.996	5	2	*pH*_*i*_, *H*_*nc*_	3	*K*^0^,*α*_*c*_, *P*	0	----
76	52 Ser*	3.247 ± 0.729 0.946	3.166 ± 0.598 0.983	0	0	---------	0	--------	0	----
**87**	**63 Asn**	**3.335±0.607 0.980**	**3.199 ± 0.540 0.995**	**9**	**4**	***B***_***r***_**, *μ, P***_***r***_**, *p***	**4**	***N***_***s***_**, *α ***_***n***_**, *B***_***I***_**, *V***^**0**^	**1**	***M***_***w***_
**90**	**66 Arg**	**3.318 ± 0.635 0.974**	**3.191 ± 0.555 0.992**	**16**	**9**	**B**_***r***_**, R**_***F***_**, *pK'*, *pH***_***i***_**, E**_***I***_**, R**_***a***_**, H**_***p***_**, H**_***t***_**, *E***_***t***_	**7**	***F*, *M***_***v***_**, *α ***_***c*,**_**N**_***s***_**, P ^_β_^, B**_***I***_**, *P***	**0**	**----**
106	82 Lys	3.287 ± 0.680 0.960	3.178 ± 0.579 0.987	4	3	*B*_*r*_, *pH*_*i*_, *E*_*t*_	1	*N*_*s*_	0	----
**108**	**84 His**	**3.368 ± 0.550 0.995**	**3.207 ± 0.524 0.998**	**6**	**3**	***pH***_***i***_**, *H***_***nc*,**_***p***	**3**	***F*, *α ***_***c***_**, *P***	**0**	**----**
117	93 Gln	3.367 ± 0.551 0.995	3.208 ± 0.523 0.999	5	3	*B*_*r*_, *pH*_*i*_, *E*_*t*_	2	B_*I*_, *α*_*c*_	0	----
127	103Val	3.229 ± 0.75 0.936	3.150 ± 0.628 0.976	1	0	---------	1	*α*_*n*_	0	----

**ω **and Bayesian (BEB) analysis posterior probabilities obtained with the NJ topology are shown for sites with PP > 0.95 in M8 that also have a PP > 0.90 in M2a. TreeSAAP analysis results present the total number of radical changes in amino acid properties and their assigned categories. Type I sites are shown in bold. *Site 52 had only strong support when using the NJ topology.

Properties symbols are as following: ***α ***_***c***_*: *Power to be – C-term., α-helix; ***α ***_***n***_*: *Power to be in the N-terminal of an α-helix; ***B***_***r***_*: *Buriedness; ***B***_***I***_*: *Bulkiness; ***E***_***I***_*: *Long-range non-bonded energy; ***E***_***sm***_*: *Short and medium range non-bonded energy; ***E***_***t***_*: *Total non-bonding Energy; ***F****: *Mean r.m.s. fluctuation displacement; ***H****: *Hydropathy; ***H***_***nc***_*: *Normal consensus hydrophobicity; ***H***_***p***_*: *Surrounding hydrophobicity; ***H***_***t***_*: *Thermodynamic transfer hydrophobicity; ***K***^**0**^*: *Compressibility; ***μ****: *Refractive index; ***M***_***v***_*: *Molecular volume; ***M***_***w***_*: *Molecular weight; ***N***_***s***_*: *Average number of surrounding residues; ***P***_α_*: *α- helical tendencies; ***P***_β_*: *β-structure tendencies; ***P***_***c***_*: *Coil tendencies; ***P****: *Turn tendencies; ***p****: *Polarity; ***pH***_***i***_*: *Isoelectric point; ***pK'****: *Equilibrium Constant of ionization for COOH; ***P***_***r***_*: *Polar requirement; ***R***_***a***_*: *Solvent accessible reduction ratio; ***R***_***F***_*: *Chromatographic index; ***V***^**0**^*:*Partial specific volume;

Some concerns have been raised over the reliability of particular sites inferred to be under positive selection using PAML [36]. Further support for the PAML results was obtained using a complementary protein level approach implemented in TreeSAAP [37]. This program performs ancestral sequence reconstruction to determine and categorize evolutionary changes in 30 amino acid properties. The number of radical changes per site was used as an indicator of positive selection. An empirical threshold of six properties with radical changes was adopted to further support previous candidate sites. Most of the 15 PAML sites had radical amino acid changes, which in five cases had a number of properties above the defined threshold (n ≥ 6) (table 3). Site 66 had the highest number of properties under selection (n = 16). In order to facilitate posterior analyses, a categorization was introduced: sites that were above the defined threshold in TreeSAAP were designated as type I, whereas the remaining sites were designated as type II.

**Figure 2:**
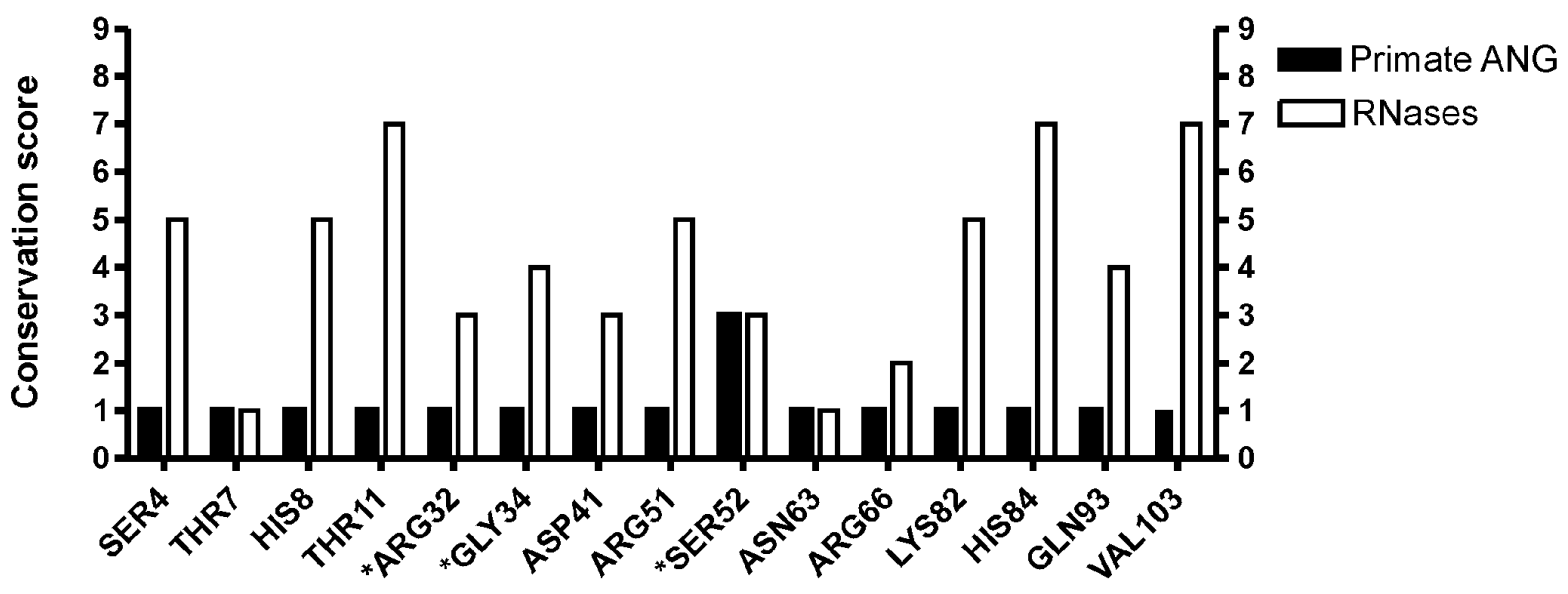
**ConSurf conservation scores for sites under positive selection**. Comparison of ConSurf conservation scores for primate angiogenin protein sequences and a pool of 168 non-angiogenin RNase sequences. (*) Indicates sites that were below the confidence cut-off for this analysis.

In order to assess if the sites under selection were only variant in angiogenin or throughout the whole RNase A superfamily, the primate ANG sequences were compared with a pool of 168 non-angiogenin RNase sequences using the ConSurf web server [38]. This software calculates evolutionary conservation scores (1 to 9) based on alignments and a reference structure (human ANG 3D-structure) (figure 2). The sites under positive selection in the primate sequences presented low conservation scores of 1, except for site 52 that had a score of 3. Conservation scores for sites 32, 34 and 52 were below the confidence cut-off for ConSurf. When analyzed in the pool of RNase sequences, sites 11, 84 and 103 presented a high conservation score of 7; whereas sites 4, 8, 51 and 34, 93 had moderate conservation scores of 5 and 4, respectively. The remaining sites had lower scores. It is striking that eight of the 15 sites detected under selection in the primate ANG gene, including three of the five type I sites, are highly or moderately conserved in the RNase A family. Although this might result in part from the structural and functional divergence between the members of this family, it also indicates that these sites are not subject to random variation throughout the family as a result of structural and functional permissiveness on their locations.

### Structure-function analyses

**Figure 3:**
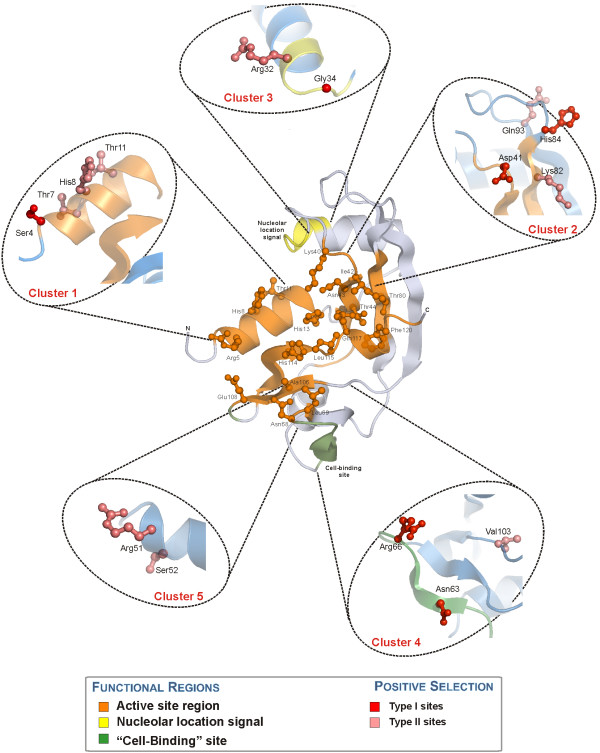
**Map of sites under positive selection and ANG functional regions**. The three main functional regions of ANG are represented in the centre image: (i) the ribonucleolytic active site (as determined by superimposition with RNase A [42]) with the main functional amino acid side chains depicted as ball and stick; (ii) the nucleolar location signal and (iii) the "Cell-Binding" site. Lateral images highlight the five structural clusters of sites under positive selection (determined using PAML and TreeSAAP), four of which are located within or in the vicinity of the three main functional regions.

To envisage possible structure-function implications of positive selected sites in the ANG gene, the candidate sites were mapped on the ANG X-ray structure (figure 3). Sites clustered in particular regions of the protein structure, a good indication of a non-random event. Four of the five positive selection clusters were located within known ANG functional regions: clusters 1 and 2 in the active site region, cluster 3 in the nuclear location signal and cluster 4 in the 'cell-binding' site. Interestingly, positive selection appears to act in all ANG's regions currently deemed essential for its angiogenic function. In order to obtain further insights into the structural variations of these regions, homology 3D-structure models were produced for all the ancestral and current ANG sequences, using SWISS-MODEL/Deep-view [39].

#### The active site

RNase' A active sites can be divided in different subsites corresponding to the binding sites of the phosphate (P_0_-P_n_), base (B_0_-B_n_) and ribose (R_0_-R_n_) moieties of each RNA nucleotide (reviewed in [40]). The most important ones are subsites: (1) P_1_, where scission of the P-O5' bond occurs; (2) B1, where the 3' base binds and (3) B_2 _where interaction with the 5' base occurs. Structural comparisons between RNase A and ANG have allowed the mapping and characterization of the different catalytic subsites [41,42]. Only the P_1 _subsite (containing the catalytic triad His-Lys-His) appears to be well conserved, the most striking structural differences residing in the B_1 _subsite that appears to be blocked by Gln117 and partly by Phe120.

As expected due to functional constraints, no evidence for positive selection was detected in amino acids with important roles to the P_1_, B_1 _or B_2 _catalytic subsites. Cluster-1 of positive selected sites under positive selection was located in the subsite P_2 _region, and included type I site, 4 (Ser) and three type II sites: 7 (Thr), 8 (His) and 11 (Thr). These sites were all located within the first ANG α-helix, neighboring several conserved important amino acids. Site 4 presented the greatest diversity of drastic changes in chemical and structural properties, linked to polarity, hydrophobicity, bulkiness and structural conformation. A more reduced diversity was obtained for site 7 mostly reflecting chemical changes, whereas sites 8 and 11 had few and mostly conformational properties under positive selection.

**Figure 4:**
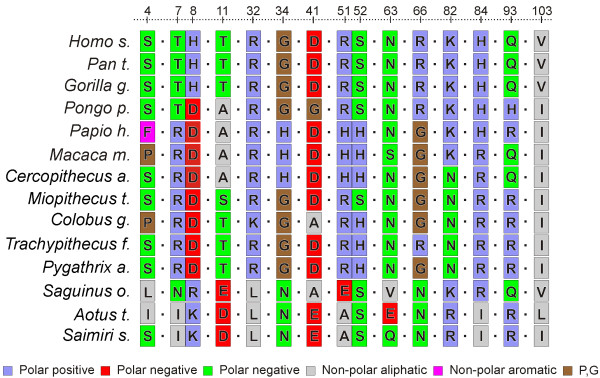
Multiple sequence alignment of amino acid sequences for sites under positive selection.

**Figure 5:**
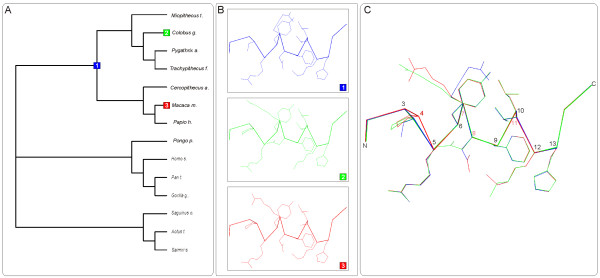
**C_α _trace and relevant side chains of 3-D structure models at the cluster-1 region**. Phylogenetic tree obtained from ancestral reconstruction in PAML (A) and superimposed 3D models for colored nodes and species (B), represented as Cα trace with the relevant side chains visible. Each amino acid is numbered in colors according to the site category: type I sites are numbered in red and type II in pink; other sites are shown in black.

Interestingly, cluster-1 presented a proline in site 4 for two of the primate species analyzed, *Colobus guereza *and *Macaca mulatta *(figure 4). This amino acid may cause the kinking of α-helices (reviewed in [43]) thereby affecting the local structure of this region. To further evaluate the effects of these substitutions, we compared the 3D-structure models for these two species with the 3D-strucutre model inferred for the most recent common ancestral of these, superimposing the protein backbones (figure 5). *Macaca mulatta *ANG presented a significant backbone distortion that was not so pronounced in *Colobus guereza *ANG. The overall structure of this region does not appear to be significantly affected, given the good positional overlap between the side chains of critical amino acids like neighboring Arg5 and also Phe9 and His13. The location of site 4 at the beginning of the helix is perhaps allowing some distortion without significantly affecting the position of critical amino acids.

Cluster-2 included four selected sites, type I sites 41 (Asp) and 84 (His), type II sites 82 (Lys) and 93 (Gln), located close or within the anti-parallel β-sheet formed by β-strands B1, B4 and B5. Sites 41 and 82 lie at the C-terminal region of the active site, while sites 84 and 93 are located further up, facing the exterior of the protein. Site 41, neighboring two important active site residues Lys40 [P_1 _subsite] and Ile42 [B_1 _subsite] (table 4), presented a total of seven amino acid properties suffering drastic changes, namely properties pertaining polarity and size/molecular weight. As for site 82, located close to another residue of the B1 subsite Thr80, four properties were detected concerning mostly the acid-base characteristics and the buriedness of the residue. The second site under positive selection in strand B4, site 84, presented a total of six properties under selection related with polarity, acid-base properties and conformational properties. Finally, site 93 (located between two important structural sites Cys92 and Tyr94; table 4) had a total of five properties detected pertaining to acid-base, size and conformation-related characteristics.

**Table 4:** Functional information for sites within or neighboring positive selection clusters

**Site**	**Functional information**		**Selection**
Ser4	None		++
Arg5	•Conserved site, unique to ANG (RNase counterpart is Ala4), has been implicated in the formation of hydrogen bonds with the P_2 _phosphate and appears to be the critical residue in this subsite [42] [45] [76]. •Involved in the contacts of the complex ANG-Ribonuclease Inhibitor (RI) [77].		
Thr7	None		+
His8	•Structural counterpart of the RNase A P_2 _subsite residue Lys7 lays 4.5Å apart from the P_2 _phosphate group in superimposed structures, too far to interact with it. Forms H-bonds with Arg33 [42]. •Involved in the contacts of the complex ANG-RI [77].		+
Phe9	•Part of the hydrophobic nucleus. Forms π-π interactions with catalytic residue His13 [78].		
Leu10	•Mutation to proline disrupts ribonucleolytic activity (disrupts interaction 9–13) [78].		
Thr11	•Structural counterpart of the RNase A P_2 _subsite residue Arg10, but lays too far from the P_2 _phosphate group in superimposed structures to form interactions [42].		+
Gln12	•Structural analogue of the RNase A P_1 _subsite residue Gln11 [42]. •Mutation to proline disrupts ribonucleolytic activity (disrupts interaction 9–13) [78]. •Mutation to Leu found in two ALS patients of Scottish/Irish descent [13].		
His13	•Member of the catalytic triad – general base catalysis [42].		
Arg31	Region 31–35 constitutes a nuclear location signal responsible for the nucleolar location of angiogenin [20].	•Mutation to alanine significantly reduces nuclear translocation efficiency. •Involved in the contacts of the complex ANG-RI [77]. •Mutation to Leu found in one ALS patients of Irish/English descent [13].	
Arg32		•Involved in the contacts of the complex ANG-RI [77].	+
Arg33		•Mutation to alanine disrupts nuclear translocation [20] and reduces ribonucleolytic activity by 7 fold [45]. •Interacts with Phe45 and shields Met30 and Cys26 from solvent [78]. •H bonds with Thr11 and Tyr14 and Ser28 [42].	
Gly34			++
Leu35			
Lys40	•Member of the catalytic triad – donates H bond to the pentavalent transition state. Conservative replacement with arginine causes a 50 fold reduction in activity [79]. •Involved in the contacts of the complex ANG-RI [77]. •Mutation to Ile found in three ALS patients of Irish/Scottish descent [13].		
Asp41	•Involved in the contacts of the complex ANG-RI [77].		++
Ile42	•Structural counterpart of the RNase A B_1 _subsite residue Val43 [42].		
Asn43	•Structural counterpart of the RNase A B1 subsite residue Asn44 [42].		
Arg51	•Flexible residue [42]. •Appears disordered in the ANG–RI complex x-ray structure [77].		+
Ser52	•Forms H-bonds with Asn61 [42].		+
Ile53	•Part of the hydrophobic core, essential to the ribonucleolytic activity [78].		
Asn61	The region from Lys-60 to Asn-68 constitutes a critical cell-binding site, distinct from the catalytic site [18].	•Deamination abolishes angiogenic activity [19]. •Conserved throughout angiogenins, considered essential for actin binding [48]. •Forms H-bonds with Ser 52 and 74 [42].	
Gly62		•Conserved throughout angiogenins, considered essential for actin binding [48].	
Asn63		•Forms H-bonds with Gly62 [42].	++
Pro64			
His65			
Arg66			++
Glu67		•Involved in the contacts of the complex ANG-RI [77].	
Thr80	•Structural counterpart of the RNase A B1 subsite residue Asp83 [42].		
Lys82	None		+
His84	•The region 84–89 is involved in the contacts of the complex ANG-RI [77].		++
Cys92	•Forms an S-S bond with Cys39 [42].		
Gln93	•Involved in the contacts of the complex ANG-RI [77].		+
Tyr94	•Forms H-bonds with Lys-40 and is part of the hydrophobic core, mutation to asparagine abolishes ribonucleolytic activity [78].		
Val103	•Part of the hydrophobic core, mutation to Asp abolishes ribonucleolytic activity [78].		+

Sites under positive selection are indicated in the last column, (++) type I sites, (+) type II sites.

Overall, the results for the two clusters located in the active site region suggest that selective forces act in several of the more permissive sites of subsites P_2 _and B1, shaping the local chemical and conformational environment without significantly interfering with the position critical residues, which are probably subject to purifying selection.

#### The nucleolar targeting sequence

The nucleolar import of ANG was mapped in the position ^31^RRRGL^35 ^[20], a region encompassing the c-terminal region of helix H2 and the beginning of loop L2. ANG import was further shown to be independent of the classic nuclear localization signal-importin α/β pathway with a proposed mechanism involving import through passive diffusion and retention in the nucleus/nucleolus mediated by the NTS region [44].

Cluster-3 of sites under positive selection is located in this region with a type I site 34 (Gly) and a type II site 32 (Arg). Analysis of drastic changes in amino acid properties for site 32 resulted in a total of five properties indicative of changes in polarity and helical conformation. Site 34 had an elevated number of drastic changes in properties as a result of the broad diversity of amino acid substitutions (figure 4). The chemical and structural divergence observed for these two sites will surely influence the interactions with other proteins mediated through this region. However, further clarification of the pathway for nuclear import or retention and the amino acid sites involved in both partners would be required in order to fully assess the impact and possible causes for positive selection in this region.

#### The cell-binding site

A putative 'cell-binding' site was first mapped to the region between residues 60 and 68, as the proteolytic cleavage of peptide bonds 60/61, 67/68 or both, abolished the angiogenic activity without significantly affecting the enzymatic activity [18]. Mutation of Arg66 [45] and the substitution of ANG residues 58–70 for their RNase counterparts produced similar results. Deamination experiments pointed out a second important residue, Asn61, and also a possible second region containing Asn109 [19]. The 'cell-binding' site was implicated in the binding to α-actin [46,47], in particular residues Asn61 and Gly62, conserved in angiogenins [48]. However, the interacting region remains elusive in other identified interacting proteins as a putative 170 kDa receptor [49] or α-2-actinin [50].

Cluster-4 of sites under positive selection partly overlaps with the cell-binding site, including two type I sites: 63 (Asn) and Arg66 and a type II site: Val103. For the first of these sites, a total of nine properties were subjected to drastic changes: four chemical properties, four structural properties and one uncategorized property. These are mostly related to polarity and volume/spatial occupation. Site 66 presented the highest number of properties subject to drastic changes in this analysis with a total of 16 out of 31 properties – nine chemical and seven structural. Overall, the evolutionary changes observed for sites 63 and 66 influence the interactions performed by this region. However, further information about the interactions between this region of ANG and other proteins would be required in order to further evaluate the effects of positive selection in this region.

Site 103 (Val) is a buried residue, but was also included in this cluster, since its α-carbon is relatively close to sites 63 and 66 (93–63: 10.84 Å; 93–66: 12.84 Å; 63–66:9.98 Å). This site is part of the hydrophobic core of the enzyme (table 4) and only one structural property was detected. The observed amino acid changes (figure 4) were replacements between branched-chain amino acids, conservative in terms of hydrophobicity.

#### Clusters outside know functional regions

Cluster-5 had two type II sites, 51 (Arg) and 52 (Ser), located in helix 3 outside of the main functional regions. The support for site 52 as being under selection was weak, only obtained with the NJ topology in PAML. As for site 51 no particular functional or structural information was available, besides being a rather flexible residue (table 4) that is exposed to the solvent. It has a total of five properties with drastic changes, mostly concerning acid-base and conformational characteristics.

### Driving forces for diversifying selection

Previous work by *Zhang and Rosenberg *[29] had reported positive selection in the primate ANG gene and analyzed its effects in terms of charge-altering substitutions. In this study we present a more extensive analysis at the gene and protein level and obtain evidence for positive selection in all the ANG regions considered essential for angiogenesis, a good indication that this is the main underlying process for diversifying selection in this gene. However, it remains elusive on whether it is physiological or pathological angiogenesis that drives ANG evolution.

Two events are generally used as reference for physiologic and pathologic angiogenesis: placentation and cancer. All the primate groups analyzed in this study have hemochorial placentas, the most invasive form of placentation, where a direct contact between the placental and maternal circulations is established. Expression studies are only available for human placentas and indicate significant increases in the ANG levels in last trimester placentas, correlating well with the dramatic increase in placental vessel density and in fetal growth that occurs in this period [51]. Further ANG expression studies on other primate placentas, in particular those of lemurs and lorises that have non-invasive epithelichorial placentation could help to evaluate the existence of a connection between ANG levels, placentation type and positive selection on this gene. Even if such a connection is established, the apparent increasing gradient of placentation invasiveness over the primate phylogeny must be seen with caution, given that studies on mammalian placenta evolution [52,53] have shown that the *Eurtherian *ancestral already had a deeply invasive placenta and that the different forms of placentation currently observed were the result of clade-specific or convergent evolution.

As for cancer, the overexpression of ANG has been documented in many tumors and appears to correlate well with disease establishment, progression and in some cases aggressiveness (reviewed in [3]). Many authors suggest that tumor cells might increase ANG (and other angiogenic factors) expression to guarantee the blood supply of the growing tumor, however, there is little information on the genetic changes underlying this overexpression and mutations in the ANG gene predisposing to cancer are unknown.

It is noteworthy that invasive placentation and cancer progression share many features, like the invasive cell phenotype, vessel reorganization and neovascularization at the site of implantation. Therefore, any evolutionary changes that impact placentation are likely to have implications in cancer. Previously, *Zhang and Rosenberg *[29] suggested, based on the importance of ANG in pregnancy to embryo vascularization, that diversifying selection could result from an evolutionary 'conflict of interests' between mothers and fetuses. The same authors also compared ANG evolution to another cancer-related protein under positive selection BRCA1 [54], and suggested that the selective pressures acting in these genes were more likely related with the physiological functions of their encoded proteins and not with cancer. The hypothesis of evolutionary conflict was recently analyzed by *Crespi and Summers *in relation to cancer [55]. These authors suggested that the particular incidence of positive selection in cancer-related genes is motivated by the strong ongoing selection generated by evolutionary conflict (antagonistic coevolution). This hypothesis is based on the fact that the physiological processes that involve antagonistic coevolution, like resource acquisition and use, cellular replication and tissue growth are also critical to cancer predisposition. The strong selection due to antagonistic coevolution would drive the changes in conflict-related genes, and the pleiotropic effects of these changes would increase cancer risk.

The association between ANG mutations and disease has only recently been observed in amyotrophic lateral sclerosis (ALS) patients [13], mostly from Irish and Scottish descent. Seven different mutations where described in 15 individuals with both sporadic and familial forms of the disease. These affected mostly conserved amino acids of functional or structural importance like Arg31Lys(NLS), Cys39Trp (S-S bond) or catalytic Lys40Ile (table 4), that should result in moderate or severe impairment of ANG function and therefore did not coincide with any of the sites found to be under positive selection. The implication of ANG in this disease is still obscure, given that these mutations are rare, inexistent in other populations [56] and, in fact, a moderate increase in ANG expression has been documented in ALS patients [57].

Although angiogenesis appears to take centre stage as a driving force for positive selection in the ANG gene, it is still possible that there are other processes contributing to selective pressures. Several lines of evidence have accumulated supporting the existence of other ANG functions: (i) the expression of ANG mRNA is low in the developing fetus, reaching maximum levels in the adult, a pattern not temporarily related to vessel development [58] and has a widespread pattern of expression in many human cell types [59]; (ii) ANG is a component of normal serum that is upregulated in acute phase responses, suggesting an involvement in host injury response [7]; (iii) human ANG antimicrobial activity was also demonstrated *in vitro *[11,12], but it is still elusive whether this is a result of the ribonucleolytic activity or some other effect. The possible ANG involvement in injury response and innate immunity constitutes another intriguing hypothesis for a selection driving force. The constant "arms-race" between host and pathogens is a well known for maintaining a strong selective pressure in immunity-related genes. A similar possibility was raised in the mouse [24], whose six paralogous ANG genes were shown to be under positive selection, two of which presenting an antimicrobial activity (ANG1 and ANG4). The role of ANG4 in gut innate immunity was characterized *in vivo *[11]. Further characterization of human/primate ANG anti-microbial effects is required in order to assess this hypothesis. Nonetheless, this is yet another possibility that is compatible with the theory of evolutionary conflict.

**Figure 6:**
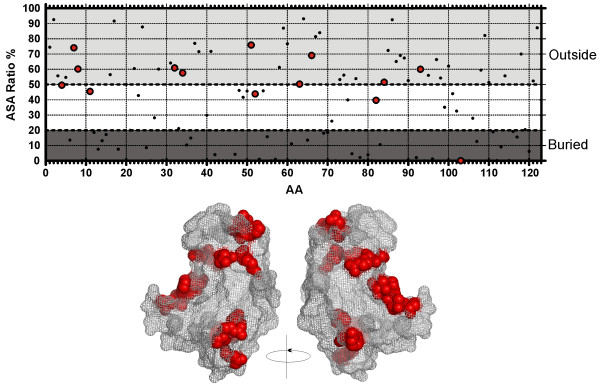
**Exposure of residues to the exterior of the protein**. Plot of the ASA ratio calculated between the side-chain and the 'random coil' value of each residue. Sites with a ratio above 50% are considered to be exposed to the exterior whereas sites under 20% are considered buried. The localization for sites under positive selection is shown in red on a wireframe representation of ANG.

Finally, it is also important to consider the role that interacting proteins and ligands may have in the evolution of ANG. We evaluated the solvent exposure of the residues detected to be under positive selection by analyzing the accessible surface area (ASA) using GETAREA (figure 6). This program estimates if a residue is exposed at the surface or buried in the protein, based on the ratio between side-chain ASA and the "random coil" values per residue. Eleven of the fifteen sites presented an ASA ratio equal or above 50% (7, 8, 32, 34, 41, 51, 63, 66, 84, and 93), three above 40% (4, 11, and 52) and only one bellow 20% (103). The majority of the sites is therefore exposed to the exterior of the protein and can *a priori *modulate the interactions with other proteins or ligands. It is also noteworthy that some of the sites detected under positive selection in this study were found to be involved in contacts with the ribonuclease inhibitor (RI) in the crystal structure of the ANG-RI complex (table 4). Furthermore, ANG has been shown to stimulate rRNA production [60], possibly mediated by binding to a specific rDNA non-transcribed sequence [61]. More precise structural characterizations of the interactions performed by ANG and its partners are still lacking and will be essential to further assess which are the critical amino acids and regions of the protein.

## Conclusion

In this work we aimed at evaluating in detail the amino acid sites under positive selection in the primate ANG gene, including its possible structural and functional impacts. Using a maximum-likelihood gene level analysis we obtained evidence for positive selection on the ANG gene. Posterior site specific analyses allowed the identification of 15 sites with strong evidence of positive selection, further corroborated by a protein level analysis that showed that five of these sites also had an elevated number of amino acid properties suffering radical changes. The mapping of these sites in the ANG 3D-structure revealed five clusters in specific regions within the main functional regions of the protein. In the active site region, positive selection appears to modulate the chemical and structural characteristics of a few permissive sites without affecting the position of the critical residues. Significant chemical and structural divergence is further observed in two other regions, the nucleolar location signal and 'cell-binding' site, clearly having a potential to influence the interactions performed through them.

Given that evidence of positive selection was detected in all ANG functional regions required for angiogenesis, it is tempting to indicate angiogenesis as the process underlying selection. It is unclear, however, if it is physiologic or pathologic involvement of ANG in angiogenesis that dictates the selective pressures. The two prototypical situations, placentation and cancer, share common features in the primate species analyzed and the hypothesis of selective pressures motivated by antagonistic coevolution appears to set a common ground explanation on how the evolutionary changes motivated by physiologic processes involving ANG can lead to an increased risk of cancer. However, other processes may also influence ANG's adaptative evolution, and the possible involvement in innate immunity is particularly interesting since the host-pathogen 'arms-race' is a common origin for selective pressure. Also, one must consider the possibility of ANG/ligands co-evolution given that most sites under selection are exposed in the surface of the protein and can mediate interactions. Future experimental characterizations of ANG function, physiologic mechanism and interactions will allow further evaluation of these hypotheses.

## Methods

### Sequence data

ANG coding sequences were retrieved from GenBank for 14 different species representative of the two main branches of the simian primates: *Platyrrhini *(new world monkeys) and *Catarrhini *(old world monkeys and hominids). Reference sequences were available for *Homo sapiens *and *Pan troglodytes*, the remaining primate sequences had been previously published [29,31] (see table 1 for species and sequence reference numbers). In all alignment and figures the names of the species were abbreviated as the genus plus the first initial of the specific epithet and the human amino acid sequence was used as reference for sites.

### Sequence alignments and phylogenetic trees

A protein based coding sequence alignment was constructed by aligning translated protein sequences using the Clustal W algorithm [62] with default settings, in MEGA version 3.1 [63] and retrieving the corresponding DNA sequence. The alignment was straightforward, with the introduction of a single 3 bp gap corresponding to an insertion of an arginine residue in the Hominid lineage. Gaps were removed from analyses. Phylogenetic trees were constructed using three distinct algorithms: neighbor-joining (NJ) [64] with 1,000 bootstrap replicates [65] in MEGA version 3.1; Maximum likelihood (ML) in Paup 4.0b10 [66] using PaupUp graphical interface [67] and Bayesian analysis (BY) in MrBayes 3.1.2 [68]. For ML the best substitution model was evaluated using Modeltest 3.7 [69] that determined SYM + G as the best-fit model, according to Akaike's information criterion (AIC). The best phylogenetic tree was determined using heuristic search with nearest-neighbor interchange (NNI) and nodes support was evaluated by bootstrapping with 1,000 replicates. For Bayesian analysis the best substitution model was evaluated through MrModelTest v2.2 [70], a modified version of David Posada's Modeltest 3.6 rewritten to compare all of the 24 models that can be implemented in MrBayes version 3, which also selected SYM+G as the best-fit model (AIC).

### Evolutionary analyses

Alignments and the NJ/ML/BY trees were used for posterior molecular evolution analyses. Evidence for positive selection on ANG was first evaluated using likelihood ratio tests using the CODEML algorithm of the PAML 3.14b package [33]. We tested Branch models, the most simple (one ratio) of which admits a single ω ratio for the entire tree and the most general (free-ratios) which allows a ω ratio for each branch. We also tested two ratios models allowing a background ω ratio and a different ω for the branch being tested, done for all the branches presenting a ω > 1 in the free-ratios models. As null hypothesis we used the one ratios model and two ratios models with a fixed ω = 1 in the branch under analysis. The level of significance for these LRTs was calculated using a chi-square approximation given that twice the difference of log likelihood between the models (2ΔlnL) will asymptotically have a χ^2 ^distribution, with a number of degrees of freedom corresponding to the difference of parameters between the nested models. We then used site models that compare the fit of two nested site specific models to the data – a neutral model that does not admit positive selection (ω < = 1) and a more general, alternative model that admits positive selection (ω > 1). The one ratio model (M0) and three pairs of site specific models were used, as suggested in the PAML user's guide: M1a (NearlyNeutral) versus M2a (PositiveSelection); M7 (Beta) versus M8 (Beta&ω) and M8a (Beta&ω_s _= 1) versus M8 (Beta&ω). The significance of the LRT between the neutral and alternative model was assessed as described before and due to possible complications with non-estimable parameters, the following degrees of freedom were used, as they are expected to be conservative [58]: M1a vs. M2a df = 2; M7 vs. M8 df = 2; M8a vs. M8 df = 1. Similar results were obtained for all methods of tree reconstruction, reflecting the robustness of PAML in respect to the phylogenetic tree used.

A protein level analysis was performed using TreeSAAP 3.2 [37]. This program calculates the goodness-of-fit between an observed distribution of changes in amino acid physiochemical properties and an expected distribution that each amino acid replacement is equally likely under selective neutrality. These are analyzed based on the ancestral reconstruction inferred from the coding sequence alignment and corresponding phylogenetic tree using the CODEML algorithm. The program categorizes the range of changes in amino acid properties in eight magnitude categories from conservative to radical and calculates a z-score that indicates the direction of selection. We chose to monitor positive radical variations (+6, +7 and +8. magnitudes) as they are expected to result in significant structural and functional changes on the protein, thereby correlating with molecular adaptation and positive selection [71,72]. The number of properties under positive selection per site was determined by summing the number of unique properties in these magnitude categories per branch. All the 31 properties in TreeSAAP were used and, in order to facilitate functional analyses, categorized in three groups: chemical, structural and others (see additional file 3). Codon numbering is according to the coding sequence alignment. Amino acid numbering is according the coding sequence numbering minus the 24 amino acids of the signal peptide.

The conservation of the sites detected under selection in the RNase A family was tested using the ConSurf web server [38]. This program calculates conservation scores for sites in a protein, based on a sequence alignment and phylogenetic trees, through an empirical Bayesian approach. Clustal-W alignments of the 14 primates sequences used in this study and a pool of 168 non-angiogenin RNase sequences (obtained by 6 psi-blast iterations using the *Homo sapiens *sequence as query) were submitted to the server. The 2ANG pdb file was used as reference and the phylogenetic tree was the one generated by ConSurf. Confidence intervals for the conservation scores estimations are calculated and when the number of sequences is small, the confidence interval tends to be large. Amino acid positions that are assigned confidence intervals that are too large are considered to be below the confidence cut-off. This was the case for sites 32, 34 and 52 in the primate ANG alignment.

### Structural analysis and homology modeling

The main functional regions and sites under positive selection were mapped on the X-ray crystallographic structure of Human ANG, retrieved from the Protein Databank as 2ANG [40]. Visualization and editing of the structure were performed using pyMOL (DeLano Scientific, San Carlos, CA, USA). Homology modeling was performed for ancestral and current sequences, whereas the ancestral sequences were reconstructed using PAML [33]. 2ANG was used as template for Swiss-model [39] using the project mode in DeepView/Swiss-pdb viewer. Briefly, the pdb x-ray coordinates file 2ANG (without waters and heteroatoms) and the sequence to model were loaded in DeepView and the resulting alignment manually corrected. This project was then submitted to the Swiss-Model server for automated model generation and energy minimization, thereby creating the final optimized model. The model quality was assessed by What-Check [73] through Swiss-Model. Furthermore, given the high degree of homology between sequences, the quality of the models is expected to be high and depending mostly on the quality of the alignment [74].

The surface exposure of amino acids was assessed using GETAREA 1.1 [75] web interface. This program performs analytical calculation of solvent accessible surface area based on the atom coordinates of a PDB file and provides an estimate of the solvent exposure based on the ratio of the side-chain surface area to "random coil" value per residue. The "random coil" value of a residue × is the average solvent-accessible surface area of × in the tripeptide Gly-X-Gly in an ensemble of 30 random conformations. Residues are considered to be solvent exposed if the ratio value exceeds 50% and to be buried if the ratio is less than 20%

## Abbreviations

ECP – Eosinophil cationic protein

EDN – Eosinophil derived neurotoxin

LRT – Likelihood ratio test

UTR – Untranslated region

## Competing interests

The author(s) declare that there are no competing interests.

## Authors' contributions

DSO performed all phylogenetic, evolutionary and structure-function analyses and drafted the manuscript, AA participated in the genetic analyses, design, drafting and coordination of the study, MJR participated in the drafting and coordination of the study. All authors read and approved the final manuscript.

## Supplementary Material

Additional file 1 Neighbor-Joining phylogenetic tree of the primate species analyzed showing branch labeling as used in the PAML branch analyses. Click here for file

Additional file 2 Likelihood ratio tests for PAML branch models. Click here for file

Additional file 3 Categorization of TreeSAAP properties. TreeSAAP properties divided in three categories based on their nature: chemical, structural or others. Click here for file
