# Supplementary figures and images for: Structural and functional implications of positive selection at the primate angiogenin gene
Source: BMC Evol Biol. 2007 Sep 20;7:167. doi: 10.1186/1471-2148-7-167 (PMC2194721; doi:10.1186/1471-2148-7-167)

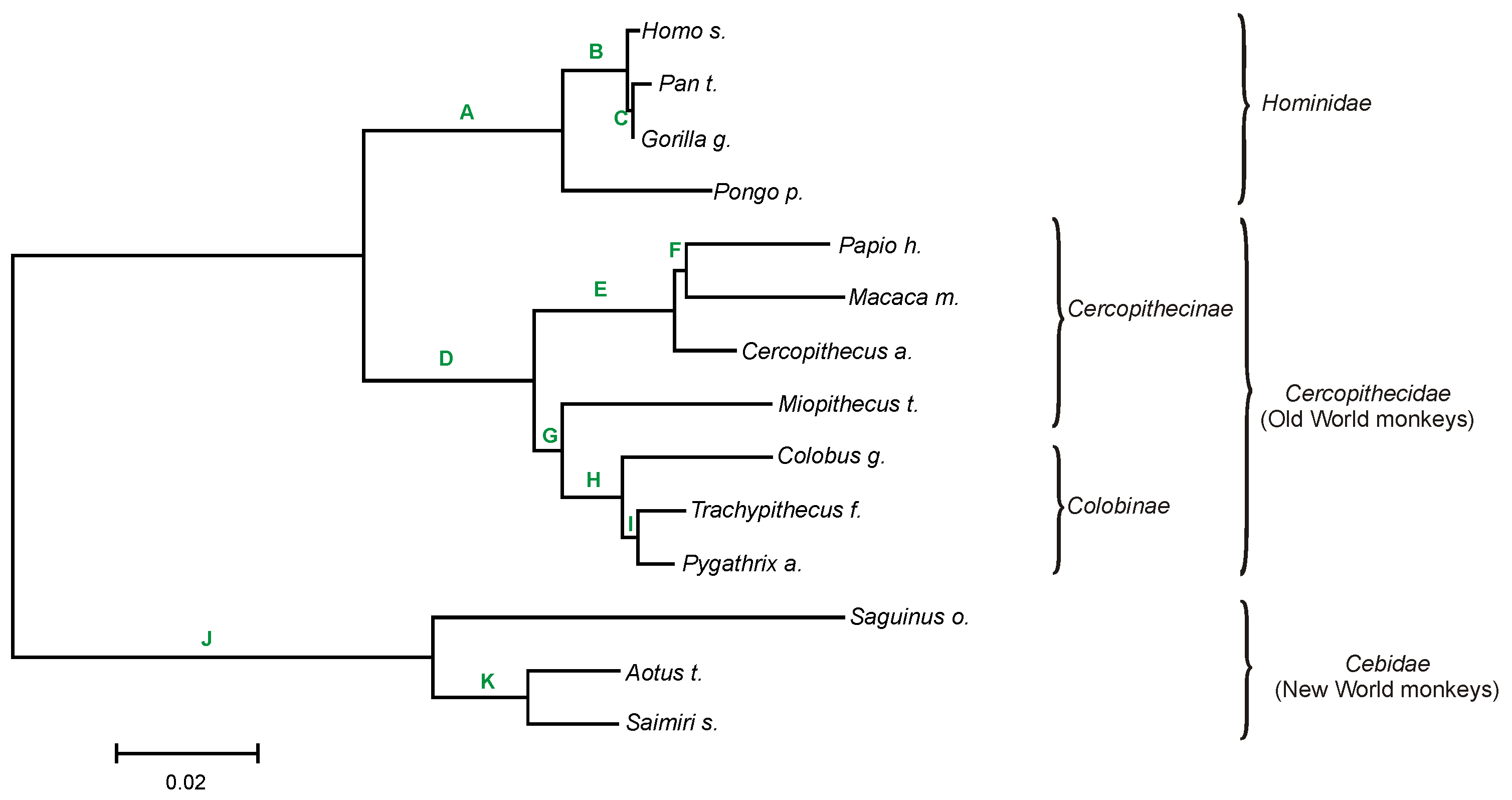

Supplement: Additional file 1 — Neighbor-Joining phylogenetic tree of the primate species analyzed showing branch labeling as used in the PAML branch analyses. [file 1471-2148-7-167-S1.png]
