# Supplementary material for: Structural and functional implications of positive selection at the primate angiogenin gene
Source: BMC Evol Biol. 2007 Sep 20;7:167. doi: 10.1186/1471-2148-7-167 (PMC2194721; doi:10.1186/1471-2148-7-167)
Supplement: Additional file 2 — Likelihood ratio tests for PAML branch models. [file 1471-2148-7-167-S2.doc]

**Additional file 2**

| **Likelihood ratio tests for PAML branch models** | | | | | | |
| --- | --- | --- | --- | --- | --- | --- |
| Model | | p | Results | LnL | **2ΔlnL (LRT)** | |
|  |  |  |  |  | A | B |
|  | |  |  |  |  |  |
| One ratio | | 27 | ω_0_=1.2934 | -1632.9385 | ----- | ------ |
| Free ratios | | 50 |  | -1618.7712 | 28.3347 (df= 23; p= 0.2034) | ----- |
| Two ratios | |  |  |  |  |  |
|  |  |  |  |  |  |  |
|  | Branch A | 28 | ω_0_=1.2744  ω_1_=1.6432 | -1632.8967 | 0.0836  (df=1; *p*=0.7725 ) | 0.3582  (df=1; *p*=0.5495) |
|  | Branch B | 28 | ω_0_=1.2614  ω_1_=∞ | -1632.3018 | 1.2735  (df=1; *p*=0.2591 ) | 1.5807  (df=1; *p*=0.2087) |
|  | Branch C | 28 | ω_0_=1.2934  ω_1_=1.0085 | -1632.9385 | 0.0000  (df=1; *p*=1.0000 ) | 0.0000  (df=1; *p*=1.0000) |
|  | Branch D | 28 | ω_0_=1.3011  ω_1_=1.2071 | -1632.9332 | 0.0106  (df=1; *p*= 0.9179) | 0.0762  (df=1; *p*=0.7825) |
|  | **Branch E** | **28** | **ω_0_=1.1943**  **ω_1_=∞** | **-1630.3217** | **5.2336 ***  **(df=1; *p*=0.0222)** | **6.2850 ***  **(df=1; *p*=0.0122)** |
|  | Branch G | 28 | ω_0_=1.2783  ω_1_=∞ | -1632.7833 | 0.3105  (df=1; *p*=0.5774) | 0.3475  (df=1; *p*=0.5556) |
|  | Branch H | 28 | ω_0_=1.2727  ω_1_=∞ | -1632.4363 | 1.0044  (df=1; *p*= 0.3163) | 1.4863  (df=1; *p*=0.2228) |
|  | Branch J | 28 | ω_0_=1.1734  ω_1_=1.9582 | -1632.3900 | 1.0969  (df=1; *p*= 0.2949) | 2.4192  (df=1; *p*=0.1199) |
|  | Branch K | 28 | ω_0_=1.2483  ω_1_=∞ | -1632.1341 | 1.6089  (df=1; *p*= 0.2047) | 1.9739  (df=1; *p*=0.1600) |
| Nine ratios  (all previous branches) | | 36 | ω_0_=1.2483  ω_A_=1.6163  ω_B_=∞  ω_C_=1.3158  ω_D_=0.8559  ω_E_=∞  ω_G_=1.1423  ω_H_=∞  ω_J_=1.9457  ω_K_=∞ | -1626.4415 | 12.9941  (df=9; *p*=0.1629) | 14.5192  (df=9; *p*=0.1050) |
| Two ratios ω_1_=1 | |  |  |  |  |  |
|  |  |  |  |  |  |  |
|  | Branch A | 27 | ω_0_=1.2782 | -1633.0758 |  |  |
|  | Branch B | 27 | ω_0_=1.2679 | -1633.0921 |  |  |
|  | Branch C | 27 | ω_0_=1.2934 | -1632.9385 |  |  |
|  | Branch D | 27 | ω_0_=1.3022 | -1632.9713 |  |  |
|  | Branch E | 27 | ω_0_=1.1892 | -1633.4642 |  |  |
|  | Branch G | 27 | ω_0_=1.2930 | -1632.9570 |  |  |
|  | Branch H | 27 | ω_0_=1.2724 | -1633.1795 |  |  |
|  | Branch J | 27 | ω_0_=1.1678 | -1633.5996 |  |  |
|  | Branch K | 27 | ω_0_=1.2640 | -1633.1210 |  |  |
| Nine ratios ω_1_=1 | | 27 | ω_0_=0.8647 | -1633.7011 |  |  |

PAML branch models admitting a single ω ratio for the whole tree, a two-ratios model allowing a different ω ratio in specific branches and a nine ratios model allowing a different ω ratio for each branch were compared with the null hypothesis of a single ω ratio or with a model where the ω for the branch under analysis is fixed to 1. The single brach with a significant LRT is shown in bold and the scores marked with (*).
