# Supplementary material for: Structural and functional implications of positive selection at the primate angiogenin gene
Source: BMC Evol Biol. 2007 Sep 20;7:167. doi: 10.1186/1471-2148-7-167 (PMC2194721; doi:10.1186/1471-2148-7-167)
Supplement: Additional file 3 — Categorization of TreeSAAP properties. TreeSAAP properties divided in three categories based on their nature: chemical, structural or others. [file 1471-2148-7-167-S3.doc]

Additional file 3

**Categorization** of TreeSAAP properties.

| **Property Name** |  | **Symbol** |  | **Category** |
| --- | --- | --- | --- | --- |
|  | | | | |
| Buriedness |  | *B_r_* |  | Chemical |
| Chromatographic index |  | *R_F_* |  | Chemical |
| Equilibrium constant of ionization -COOH |  | *pK'* |  | Chemical |
| Hydropathy |  | *h* |  | Chemical |
| Isoelectric point |  | *pH_i_* |  | Chemical |
| Long-range non-bonded energy |  | *E_I_* |  | Chemical |
| Normalized consensus hydrophobicity |  | *H_nc_* |  | Chemical |
| Polar requirement |  | *P_r_* |  | Chemical |
| Polarity |  | *p* |  | Chemical |
| Refractive index |  | *μ* |  | Chemical |
| Short and medium range non-bonded energy |  | *E_sm_* |  | Chemical |
| Solvent accessible reduction ratio |  | *R_a_* |  | Chemical |
| Surrounding hydrophobicity |  | *H_p_* |  | Chemical |
| Thermodynamic transfer hydrophobicity |  | *H_t_* |  | Chemical |
| Total non-bonded energy |  | *E_t_* |  | Chemical |
| Composition |  | *c* |  | Other |
| Molecular weight |  | *M_w_* |  | Other |
| α−helical tendencies |  | *P_α_* |  | Structural |
| Average number of surrounding residues |  | *N_s_* |  | Structural |
| β-structure tendencies |  | *P_β_* |  | Structural |
| Bulkiness |  | *B_I_* |  | Structural |
| Coil tendencies |  | *P_c_* |  | Structural |
| Compressibility |  | *K^0^* |  | Structural |
| Helical contact area |  | *C_a_* |  | Structural |
| Mean RMS fluctuation displacement |  | *F* |  | Structural |
| Molecular volume |  | *M_v_* |  | Structural |
| Partial specific volume |  | *V^0^* |  | Structural |
| Power to be at the C-terminal of an α-helix |  | *α_c_* |  | Structural |
| Power to be at the middle of an α-helix |  | *α_m_* |  | Structural |
| Power to be at the N-terminal of an α-helix |  | *α_n_* |  | Structural |
| Turn tendencies |  | *P* |  | Structural |

TreeSAAP properties divided in three groups based on their nature: chemical, structural and others.
